# Ectopic expression of *CYP24A1* circular RNA hsa_circ_0060927 in uterine leiomyomas

**DOI:** 10.1002/jcla.23114

**Published:** 2019-11-20

**Authors:** Elnaz Fazeli, Samira Piltan, Hossein Sadeghi, Milad Gholami, Ghasem Azizi‐Tabesh, Fakhrolmolook Yassaee, Reza Mirfakhraie

**Affiliations:** ^1^ Department of Medical Genetics School of Medicine Shahid Beheshti University of Medical Sciences Tehran Iran; ^2^ Department of Biochemistry and Genetics School of Medicine Arak University of Medical Sciences Arak Iran; ^3^ Department of Obstetrics and Gynecology Taleghani Hospital Shahid Beheshti University of Medical Sciences Tehran Iran; ^4^ Genomic Research Center Shahid Beheshti University of Medical Sciences Tehran Iran

**Keywords:** circular RNA, *CYP24A1*, hsa_circ_0060927, leiomyoma, *MED12*

## Abstract

**Background:**

As a novel class of non‐coding RNAs, the role of circular RNAs (circRNAs) in tumor biogenesis and progression has been proved in a number of human tumors; however, up to now, the relation between circRNAs and uterine leiomyomas (ULM) remains unclear.

**Methods:**

In this study, we have estimated the expression level of *CYP24A1* hsa_circ_0060927 in uterine leiomyoma and adjacent tissues considering the mediator complex subunit 12 gene *(MED12)* mutation profile by quantitative real‐time polymerase chain reaction (qRT‐PCRs).

**Results:**

Using Sanger sequencing method, somatic mutations in the *MED12* exon 2 were detected in 14 (35.90%) ULM samples, including 10 (71.43%) missense mutations and 4 (28.57%) in‐frame deletions. Our results revealed that hsa_circ_0060927 was ectopically expressed in 33.33% of ULM tissues; although, this expression was independent of the *MED12* mutation profile in the ULM samples.

**Conclusions:**

Present results provide primary evidence for the role of circular RNAs in the leiomyoma development; however, further studies are essential to confirm the importance of these molecules as potential biomarkers for diagnosis and/or prognosis in ULM.

## 1 INTRODUCTION

Uterine leiomyomas (ULMs), also stated as fibroids, are the most common pelvic tumors occurring in up to 70% of women of reproductive age.^1^ Disregarding their benign nature, ULMs can cause significant reproductive and gynecological complications, such as pelvic pain, heavy bleeding, preterm labor, and recurrent pregnancy loss.^2^, ^3^, ^4^ ULMs are a major reason for hysterectomy due to their several health problems; hence, there is an urgent need for novel treatments to replace surgical intervention, especially for women who desire later pregnancy.^5^ Despite increasing researches, the molecular mechanisms underlying ULMs tumorigenesis and development are poorly understood; therefore, it is essential to investigate possible molecular mechanisms that are involved in the disease development to detect potential biomarkers or novel therapeutic targets. According to the literature, leiomyomas have different genetic drivers, that the most common are rearrangements in *HMGA2 *(high mobility group AT‐hook 2), inactivation of *FH* (fumarate hydratase), and mutations in the *MED12* (mediator complex subunit 12).^6^ Among these, somatic mutations in the *MED12* exon 2 have a higher frequency in diverse populations.^7^, ^8^, ^9^ Mehine et al^6^ suggested that each genetic driver results in a different expression pattern in ULMs. Hence, molecular classification should be considered as a significant step in studying ULMs pathogenesis.

Circular RNAs (circRNAs) are covalently closed loop non‐coding RNA molecules, lacking 3’ tail and 5’cap that widely exist in eukaryotic cells. Previously, these molecules were considered as byproducts of splicing errors; however, several circRNAs have been reported to have physiological functions. There are a great number of studies that validated the important correlation of circRNAs and different cancer types, including their role in initiation, progression, and metastasis of cancer.^10^, ^11^ However, to date, the association between ULMs pathology and circRNAs remains unclear.

Recently, there has been a greater focus on the vitamin D role in developing ULMs. 1α, 25(OH)_2_D_3,_ the active form of vitamin D, is present in most of human tissues such as myometrium. Vitamin D has an anti‐tumorgenesis function and regulates the expression of many genes that play a role in cellular proliferation, differentiation, and apoptosis. *CYP24A1* (Cytochrome P450 family 24 subfamily A member 1) is a mitochondrial enzyme that is responsible for neutralizing active vitamin D.^12^, ^13^ According to the previous studies, *CYP24A1* functions as an oncogene and is upregulated in many tumors, such as ULMs 13, 14; however, there is no evidence concerning the role of *CYP24A1* related circRNAs in this disease. Due to the dysregulation of *CYP24A1* in ULMs, we hypothesized that its related circRNA, hsa_circ_0060927 may also play a role in the disease development. Hence, the aim of the present study was to explore the relationship between the hsa_circ_0060927 expression level in ULM tissues, regarding the *MED12* mutation profile.

## 2 MATERIALS AND METHODS

Seventy‐eight tissues, including 39 leiomyoma samples and 39 related adjacent tissues, were acquired from Iranian women with ULM who underwent myomectomy or hysterectomy in Taleghani hospital (Tehran, Iran). Surgery was performed within the first 10 days of the menstrual cycle for all patients. The tissue samples were first frozen in liquid nitrogen and then stored at −80°C. All participants provided written informed consent prior to study enrollment. This study was conducted in accordance with the ethical principles of the World Medical Association's Declaration of Helsinki and was approved by the ethics committee of the Shahid Beheshti University of Medical Sciences (SBMU) (Code: IR.SBMU.MSP.REC.1398.261).

### 2.1 Mutation analysis

#### 2.1.1 Patients

Genomic DNA was extracted from 30 mg of the tissues using CELL SV MINI kit (GeneAll) regarding the manufacturer procedure. The genomic DNA was amplified by PCR using two sets of specific primers (Table 1) to investigate possible mutations in exon 1, exon 2, and the flanking intron regions of the *MED12*. PCR reactions were prepared separately for each set of primers containing 1 µL genomic DNA (≥100 ng), 0.5 µL of each primer (5 pmol), 12.5 µL Taq DNA Polymerase 2X Master Mix Red (Amplicon), and 10.5 µL PCR‐grade water, in a total volume of 25 µL. The amplifications were performed in a GeneTouch thermocycler instrument using the following program: 95°C for 5′ as the initial denaturation step and then a series of 32 cycles of 95°C for 25″, 60.5°C for 30″, and 72°C for 35″. The last elongation step was performed at 72°C for 4′. Using an ABI 3730xl DNA analyzer, Sanger sequencing was performed to define genomic alterations in *MED12* gene. Mutation analysis was performed using Chromas software (version: 2.13).

**Table 1:** Primer sequences for mutation detection and qRT‐PCR

Genes	Primers	Sequences	Amplicon size (bp)
*MED12* exon1	Forward primer	GCCGTCCTCTCAACCACC	216
	Reverse primer	CGTCAGTTCATCCTCCTTCTGT	
*MED12* exon2	Forward primer	GAACGTAAGGGCCCAGCTTT	356
	Reverse primer	TCAGCCACTTAGGTTGTCCC	
hsa_circ_0060927	Forward primer	TAATACGCCTCAGGGAAGG	196
	Reverse primer	GACCATTTGTTCAGTTCGCT	
*Beta‐2‐microglobulin*	Forward primer	TGTCTTTCAGCAAGGACTGGT	143
	Reverse primer	TGCTTACATGTCTCGATCCCAC	

### 2.2 Expression analysis

**Figure 1:**
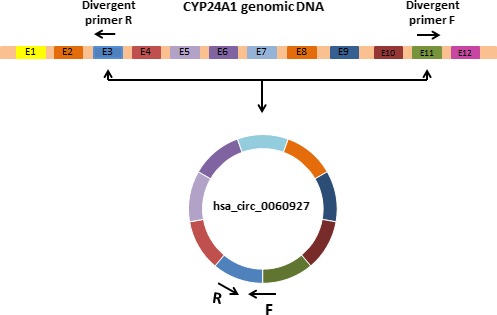
Schematic structure illustrates the biogenesis of circular RNA hsa_circ_0060927 from *CYP24A1* gene. Black arrows indicate the location of divergent primers

RNA extraction was performed from all tissue samples using GeneAll Hybrid‐R™ RNA purification kit (GeneAll Biotechnology Co. Ltd). High‐Capacity cDNA Reverse Transcription Kit was used to synthesis of the cDNA first strand from 2 μg of total RNA according to the provided protocol. The hsa_circ_0060927 sequence was obtained from the circBase online database (http://www.circbase.org/). Specific divergent primers were designed using CircPrimer software^15^ to amplify the targeted circRNA. Hsa_circ_0060927 is transcribed from *CYP24A1* and contains exons 3‐11 (Figure 1).

Quantitative RT‐PCR (qRT‐PCR) of hsa_circ_0060927 and *beta‐2‐microglobulin* (*β2M*) genes were performed in duplicate on an ABI StepOnePlus™ Real‐Time PCR Detection System. Each reaction was prepared in a total volume of 20 µL and comprised of 10 µL 2X RealQ‐PCR Master Mix^®^, 2 µL cDNA (≥10 ng), 0.5 µl of each primer (10 pmol), and 7 µL of PCR‐grade water.

The amplification program was as follows: One cycle of 94°C for 15′, and 94°C for 20″, followed by 60°C for 45″ for 40 cycles, and the melt curve stage assessment. To check the primer specificity, melting curves analysis and agarose gel (2%) electrophoresis were performed. Table 1 shows the primer sequences for the studied genes. The hsa_circ_0060927 relative expression level was normalized to the *β2M* expression level.

### 2.3 Statistical analysis

Prior to the expression analysis, the cycle threshold (*C*_t_) data and amplification efficiency for each reaction were calculated using LinRegPCR software (version: 2017.1), which calculates the amplification efficiency based on fluorescence data per cycle. The gene expression ratio (fold change) for the hsa_circ_0060927 in *MED12* mutant and *MED12* wild‐type leiomyoma samples was calculated using the REST© 2009 software (v2.0.13). We analyzed and plotted the experimental data using GraphPad Prism 8.0. Two‐tailed *t* test was used to assess the differences in the expression level of hsa_circ_0060927 in the *MED12* mutant and *MED12* wild‐type leiomyoma samples. *P* < .05 was considered statistically significant.

## 3 RESULTS

**Figure 2:**
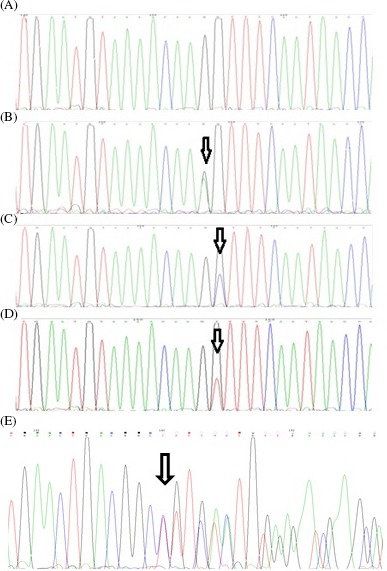
Chromatograms presenting some of the exon2‐*MED12* somatic mutations. A, Wild‐type *MED12*. B, c.130G>A. C, c.131G>C. D, c.131G>T. E, c.114_149del36

The basic characteristics of the studied patients and the ULM samples are shown in Table 2. DNA sequencing analysis revealed somatic heterozygous mutations in the *MED12* exon 2 in 14 (35.90%) ULM samples, including 10 (71.43%) missense mutations and 4 (28.57%) in‐frame deletions (Table 3) (Figure 2). No mutation was detected in the *MED12* exon 1.

**Table 2:** Basic characteristics of the studied patients and leiomyoma samples

Variable	Mean ± SD	Range
Age (y)	40.94 ± 8.78	28‐51
Body mass index (BMI)(kg/m^2^)	26.82	18.7‐36.2
Intramural myoma (%)	46.16	
Subserosal myoma (%)	38.46	
Submucosal myoma (%)	15.38	
Tumor size (cm)	4.09 ± 4.01	0.8‐20

**Table 3:** Detected *MED12* mutations in the studied uterine leiomyomas

Mutation type	Nucleotide change		Predicted amino acid change	Number of mutated samples (%)
	Genomic	Coding		
Point mutation	g.5848G>A	c.130G>A	P.G44S	5 (10.26)
	g.5848G>T	c.130G>T	p.G44C	1 (2.56)
	g.5849G>T	c.131G>C	P.G44A	1 (2.56)
	g.5849G>A	c.131G>A	p.G44D	3 (7.69)
Deletion	g.5840_5860del21	c.122_142del21	p.V41_Q48delinsE	1 (2.56)
	g.5818_5838del21	c.100_120del21	p.D34_ N40del	1 (2.56)
	g.5832_5867del36	c.114_149del36	p.A38_A50del	1 (2.56)
	g.5845_5859del15	c.127_141del15	p.Q43_N47del	1 (2.56)

We also analyzed the hsa_circ_0060927 expression level in uterine leiomyoma and adjacent tissue samples. Among leiomyoma samples, 13 samples (33.33%) showed hsa_circ_0060927 expression. Among these samples, 6 (46.15%) were positive for *MED12* mutations, and 7 (53.85%) were negative for *MED12* mutations. Leiomyoma samples positive for *MED12* mutations and ectopic expression of hsa_circ_0060927 had c.131G>A (2 samples), c.130 G>A (2 samples), c.131G>T (1 sample), and c.114_149del36 (1 sample) mutations.

**Figure 3:**
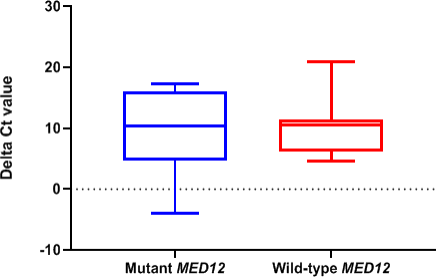
The relative expression level (Δ*C*_t_) of hsa_circ_0060927 in leiomyomas with mutant *MED12* compared to leiomyomas with wild‐type *MED12*. The difference between the expression levels was not statistically significant (*P* = .775)

The expression of hsa_circ_0060927 was not observed in the normal tissues. Expression level comparison between *MED12* mutation positive and negative leiomyoma samples showed reduced expression level of hsa_circ_0060927 in *MED12* wild‐type samples (2.04‐fold); however, the *p* value was not statistically significant (*P* = .775) (Figure 3).

## 4 DISCUSSION

Circular RNAs have gained increasing attention in recent years. Although there is little known about their role in human biological processes, the dysregulation of circRNAs is confirmed in several diseases including cancer.^11^, ^16^, ^17^ Up to now, the relation between circular RNAs and ULMs remains unclear. We evaluated the hsa_circ_0060927 expression level in uterine leiomyoma and adjacent tissue samples by qRT‐PCR. Our results showed that this circular RNA was ectopically expressed in ULM tissues. We also showed that *MED12* mutation did not have any significant effect on hsa_circ_0060927 expression (*p* value = 0.775). To our knowledge, this is the first study about the relationship between circular RNAs and ULMs. Recent studies proved hypovitaminosis D as an important risk factor in ULM; and therefore, vitamin D metabolism plays an important role in the disease development.^18^ Sharan et al^19^ showed that Vitamin D3 treatment reduces cell proliferation in immortalized ULM cells. Moreover, Eker rat model studies showed that vitamin D3 decreased the ULM size.^20^ As mentioned earlier, CYP24A1 is a mitochondrial enzyme responsible for the inactivation of vitamin D, and its overexpression is reported in ULM tissues.^13^, ^14^ Based on the circBase (http://www.circbase.org/) and two previous studies, hsa_circ_0060927 is one of the circular RNAs derived from *CYP24A1* gene.^21^, ^22^ Therefore, we hypothesized that hsa_circ_0060927 may play a role in the ULM pathology.

The mechanisms underlying the function of circRNAs are not fully understood; however, circRNAs may function as expression regulators by sponging microRNAs.^23^, ^24^ Zhong et al^24^ revealed that hsa_circ_0060927 was differentially expressed in bladder carcinoma tissue and sponged several miRNAs, including miR‐29b‐1‐5p, miR‐224‐3p, miR‐522‐3p, miR‐661, and miR‐1264. Among these miRNAs, miR‐29b affects the remodeling and production of the extracellular matrix (ECM), and its downregulation was reported in ULM cells.^25^, ^26^

Aberrant expression of miR‐224‐3p, miR‐661, and miR‐1264 play various roles in tumorigenesis, including autophagy suppression, cell differentiation, multidrug resistance, cell proliferation, and methylation induced gene silencing.^27^, ^28^, ^29^, ^30^

**Figure 4:**
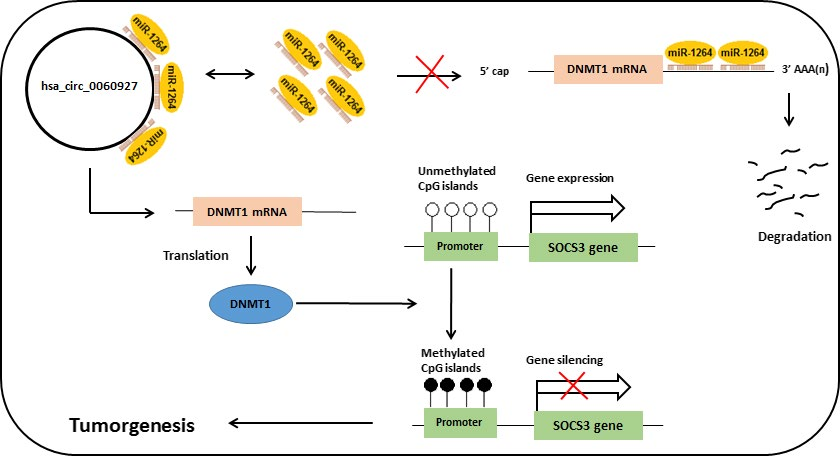
Schematic diagram representing the possible role of hsa_circ_0060927 in sponging miR‐1264 that results in the stability of DNMT1 mRNA and therefore methylation of target genes such as SOCS3

Boosani et al^30^ showed that miR‐1264 targets DNA methyltransferase‐1 (*DNMT1*), and its downregulation mediates silencing of *SOCS3* gene. DNMT1, as an important DNA methyltransferase, is responsible for the accuracy of DNA methylation pattern during DNA replication and de novo methylation.^31^, ^32^ Moreover, aberrant methylation of *SOCS3* promoter region has been reported in many human cancers.^33^ There is no previous study on the relationship between *SOCS3* and ULM yet; however, several studies reported the elevated expression of *DNMT1* in uterine leiomyoma.^34^, ^35^, ^36^ Therefore, it may be hypothesized that sponging of miR1264 by hsa_circ_0060927 results in the increase of *DNMT1* transcripts and subsequently hypermethylation of *SOCS3* gene that may contribute to ULM pathology (Figure 4).

## 5 CONCLUSION

We showed for the first time that the hsa_circ_0060927 was ectopically expressed in uterine leiomyoma compared to the adjacent tissues, and *MED12* mutation did not have any significant effect on the expression of the targeted circular RNA. Additional studies are required to clarify the exact role of hsa_circ_0060927 in ULMs. Our study may provide new visions into the molecular mechanisms involved in the ULM pathogenesis and the disease treatment.

## AUTHOR CONTRIBUTIONS

RM, FY, and MG participated in conception and design; EF, SP, and GAT involved in clinical data collection and statistical analysis; EF, SP, HS, and GAT involved in performing molecular experiments; EF, HS, MG, FY, and RM involved in drafting the article or revising critically for important intellectual content.
